# Air-Pollution-Mediated Microbial Dysbiosis in Health and Disease: Lung–Gut Axis and Beyond

**DOI:** 10.3390/jox14040086

**Published:** 2024-10-21

**Authors:** Md Habibul Hasan Mazumder, Salik Hussain

**Affiliations:** 1 Department of Physiology, Pharmacology & Toxicology, School of Medicine, West Virginia University, Morgantown, WV 26506, USA; mhm0012@mix.wvu.edu; 2 Center for Inhalation Toxicology (*iTOX*), School of Medicine, West Virginia University, Morgantown, WV 26506, USA; 3 Department of Pharmaceutical and Pharmacological Sciences, School of Pharmacy, West Virginia University, Morgantown, WV 26506, USA; 4 Department of Microbiology, School of Medicine, West Virginia University, Morgantown, WV 26506, USA

**Keywords:** air pollution, respiratory microbiome, gastrointestinal microbiome, lung–gut–liver axis, microbiome dysbiosis

## Abstract

Growing evidence suggests physiological and pathological functions of lung and gut microbiomes in various pathologies. Epidemiological and experimental data associate air pollution exposure with host microbial dysbiosis in the lungs and gut. Air pollution through increased reactive oxygen species generation, the disruption of epithelial barrier integrity, and systemic inflammation modulates microbial imbalance. Microbiome balance is crucial in regulating inflammation and metabolic pathways to maintain health. Microbiome dysbiosis is proposed as a potential mechanism for the air-pollution-induced modulation of pulmonary and systemic disorders. Microbiome-based therapeutic approaches are increasingly gaining attention and could have added value in promoting lung health. This review summarizes and discusses air-pollution-mediated microbiome alterations in the lungs and gut in humans and mice and elaborates on their role in health and disease. We discuss and summarize the current literature, highlight important mechanisms that lead to microbial dysbiosis, and elaborate on pathways that potentially link lung and lung microbiomes in the context of environmental exposures. Finally, we discuss the lung–liver–gut axis and its potential pathophysiological implications in air-pollution-mediated pathologies through microbial dysbiosis.

## 1. Introduction

According to the World Health Organization, approximately 99% of the world’s population breathes unhealthy levels of air pollutants [1]. The air-pollution-related diseases cause 7.2 million premature deaths worldwide, making air pollution a major environmental health risk [2,3]. Poor air quality is associated with idiopathic pulmonary fibrosis [4], hypersensitivity pneumonitis [5], and chronic obstructive pulmonary disease [6]. Moreover, epidemiological evidence also suggests an association between chronic gastrointestinal diseases, such as gastrointestinal cancer [7] and inflammatory bowel disease [8] and air pollution. Moreover, it has been associated with impaired respiratory function, lung development, and inflammation [9,10,11].

Air pollution is a mixture of substances, including particulate matter (PM) and gases [12]. PM is a heterogeneous mixture of metals, ions, organic matter, polycyclic aromatic hydrocarbons, sulfates, and nitrates [13]. Meanwhile, carbon dioxide, carbon monoxide, sulfur dioxide, and ozone are the most common gases in air pollution [14]. Air pollutants are mostly generated from atmospheric oxidation reactions [15], the combustion of fossil fuels [16], industrial emissions [17], and agricultural emissions [18]. Epidemiological studies demonstrate a correlation between air pollution exposure and mortality, morbidity, and a decrease in life expectancy in human cohorts [19,20,21]. Several studies demonstrate the deleterious effects of air pollution on host immunity and microbiome [13,22,23,24,25].

**Figure 1:**
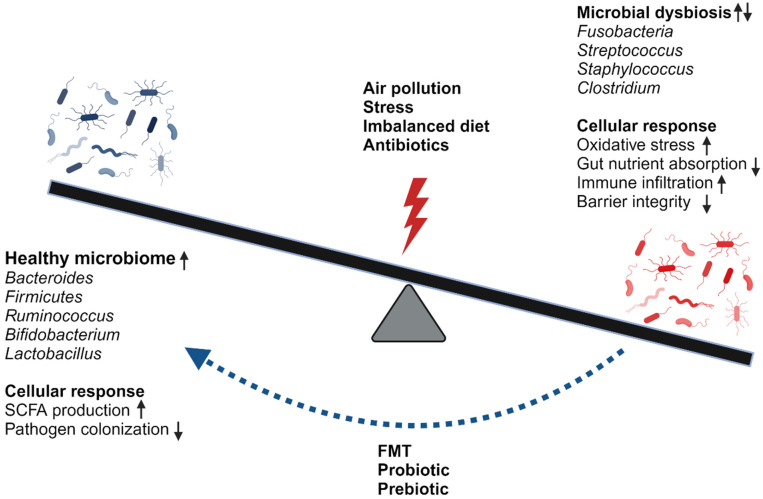
Environmental factors and host–microbiome interactions determine the fate of microbiome shift. While an increase in healthy microbiome composition suppresses pathogen invasion, dysbiosis leads to immune infiltration and disruption of tissue barrier integrity. An increase in *Ruminococcus*, *Bifidobacterium*, and *Lactobacillus* can lead to an increase in SCFA production and reduce pathogen colonization, whereas alteration in the balance of *Streptococcus*, *Staphylococcus*, and *Clostridium* impacts disease severity. Environmental factors such as air pollution, diet, and exposure to antibiotics modulate this balance and alter the cellular responses such as barrier integrity, increased neutrophilic infiltration, and increased ROS production.

Microbiota specifies the microbial community comprising bacteria, fungi, archaea, and viruses associated with the host, and the total genome of this community is known as the microbiome [26,27]. The microbiome has a crucial role in regulating inflammation and metabolic pathways, thus maintaining health. The community profiles and the composition of the microbiome differ between individuals and are influenced by environmental and host-associated factors (lifestyle, food, habitat, etc.). A delicate balance between host and microbial populations is crucial. This balance plays a vital role in the development and functioning of the immune response, growth, digestion, and neural development [28]. A shift in the balance of the microbial communities makes the host prone to pathogenic invasion and, thus, disease. Microbial dysbiosis refers to an alteration in microbial composition from optimal in a specific niche [29,30]. The composition and function of the microbiome varies based on location and disease status. The lung, which was previously thought of as a sterile organ, has a unique microbial signature compared to the gut [31]. In the lungs, microbiota regulates defense against pathogenic infiltration [32]. In the gut, microbiota helps to maintain gut immunity, motility, and intestinal barrier permeability [33,34], whereas, in the liver, microbes regulate metabolism to reduce enterotoxicity [35,36]. Several studies found that respiratory and GI tract microbiomes communicate and influence each other [37,38]. Thus, it is important to understand the communication between the lungs and the gut to alleviate pollution-mediated negative health impacts in these organs. Moreover, manipulating the microbiome to support healthy microbial composition and intestinal health could have added value in promoting lung health. Balance in microbiomes and different factors influencing microbiome dysbiosis and vice versa is presented in Figure 1.

The human microbiome comprises nearly 100 trillion microbes that are mostly bacteria and to a lesser extent protozoa, archaea, fungi, and viruses [39]. The composition of the microbiome differs depending on the site and age. *Corynebacteria*, *Streptococcus*, *Prevotella*, *Haemophilus*, *Rothia*, and *Fusobacterium* are the prominent microbiome in the oral cavity and oropharynx [40,41]. The respiratory tract microbiome is mostly comprised of *Prevotella*, *Neisseria*, *Haemophilus*, *Fusobacterium*, and *Streptococcus* [42], whereas the gut microbiome contains *Actinobacteria*, *Fusobacteria*, *Proteobacteria*, *Firmicutes*, and *Bacteroidetes* [43]. A list of bacterial communities based on the body site is presented in Table 1.

## 2. Respiratory Microbiome

The Human Microbiome Project (HMP) was started in 2007 to study the diversity, function and categories of the human microbiome [44]. Yet, upon HMP initiation, lungs were not sampled believing that healthy lungs are sterile. Metagenomics and 16 s ribosomal RNA sequencing by next-generation sequencing (NGS) led to the frequent detection of bacterial communities in lungs [45].

The respiratory tract microbiome varies with anatomy and physiology. The oral cavity contains *Prevotella*, *Streptococcus*, *Veillonella*, *Fusobacterium*, *Corynebacteria*, *Haemophilus*, and *Neisseria* [46]. The upper respiratory tract contains *Staphylococcus*, *Propionibacterium*, *Prevotella*, *Leptotrichis*, *Dolosigranulum*, *Veillonella*, *Rothia*, *Corynebacterium*, *Moraxella*, *Streptococcus*, and *Haemophilus* [47,48]. The resident upper airway microbiome is the main source of the lower airway microbiota. It has been proposed that bacteria may reach LRT through oropharyngeal secretion, micro-aspiration, or direct inhalation [49]. LRT mostly harbors *Streptococcus*, *Veillonella*, *Prevotella*, *Acidobacteria*, and *Actinobacteria* [40,50].

### 2.1. Characteristics of the Respiratory Microbiome

The microbial composition of the lungs is heterogeneous and changes in response to physiological conditions. The crosstalk between microbial immigration, elimination, and relative production determines the load of microbiome in the lungs [51,52]. In the lungs, the prominent phyla are *Firmicutes*, *Proteobacteria*, *Bacteroidetes*, and *Actinobacteria*. The most common genera are *Veillonella*, *Fusobacterium*, *Prevotella*, *Streptococcus*, *Porphyromonas*, and *Neisseria* [53]. The microbiome composition is significantly changed by the host’s disease status. Table 2 presents a summary of microbiota in different disease states.

### 2.2. Air Pollution and Respiratory Microbiome

Air pollution can have a direct (through the physical interaction of pollutants and microbes) and/or indirect impact (through the induction of inflammation and oxidant stress) on the respiratory microbiome [63]. Research findings for both human and animal models are summarized in Table 3 and Table 4. The analysis of saliva and sputum samples demonstrated higher alpha diversity indices in highly polluted areas [64]. Throat swab samples from 114 individuals demonstrated a positive correlation between polluted air and the relative abundance of *Actinobacteria* and *Proteobacteria* [65]. Another study demonstrated that the abundance of *Streptococcus* and *Neisseria* significantly increased in air-pollution-exposed subjects [66]. Indoor/household air pollution reduced alpha diversity and *Betaproteobacteria* abundance and increased *Fusobacteria* in the sputum microbiome [67]. Controlled ozone exposure in young adults reduced alpha diversity and the abundance of *Actinobacteria* and *Firmicutes*, whereas it increased *Moraxellaceae* and *Pseudomonadaceae* [68]. This demonstrates a complex relationship between pollution and microbial changes in the host. An increased level of PM correlates with the increased relative abundance of *Staphylococcus*, *Hemophilus*, *Streptococcus*, and *Moraxella* [69]. Additionally, greater bacterial load is associated with impaired respiratory function and with increases in *Streptococcus*, *Prevotella*, *Neisseria*, and *Fusobacterium* [70]. In another study, PM_2.5_ and PM_10_ exposure in healthy subjects was inversely correlated with Shannon, Chao1, and PD_Whole_Tree alpha diversity indices and reduced the abundance of *Actinobacteria* and *Proteobacteria* [71]. In lung cancer patients, a low alpha diversity correlated with low PM_10_, while *Legionella* and *Thermus* abundance had a positive association with PM_10_ exposure [72]. Moreover, increased bacterial abundance was found in the saliva samples of asthmatic children. Additionally, a significant negative association between microbial indices (species richness and observed species) and short-term PM_2.5_ or ozone exposure in asthmatic children was reported [73]. These studies demonstrated that alteration in the microbial composition depends on several factors such as host site, the type of exposure, the route of exposure, etc.

Several animal models were also investigated in addition to human studies to evaluate the effect of air pollution on the respiratory microbiome. A reduced Shannon, Fisher, and observed ASV index, a higher abundance of *Firmicutes*, and reduced *Bacteroidetes* and *Fusobacteria* were observed in the bronchoalveolar lavage from PM_2.5_ exposed Balb/c male mice [76]. Ambient PM_2.5_ exposure resulted in a decreased *Lachnospiraceae* and *Psuedomonadaceae* in the lungs of nuclear factor E2-related factor 2 knock-out (Nrf-2 KO) mice [77]. A similar study on traffic-generated emissions also found an increase in *Proteobacteria* in the lung homogenates and a decrease in the alpha diversity index [78,79].

In contrast, PM_2.5_ exposure in mice increased alpha diversity, *Bacteroidetes*, and *Cyanobacteria* abundance. In addition, alterations in serum and BALF cytokine levels (*IL-17*, *IL-6*, *IL-1b*, and *TNF-alpha*) were also reported [77]. Similarly, an increase in bacterial relative abundance, Chao1, observed species index, and *Proteobacteria* abundance have also been reported in rat lungs [80]. Daniel et al. reported an increase in *Proteobacteria* and a reduction in *Firmicutes* and *Bacteroidetes* in a model of DEP combined with diet manipulation [78]. Collectively, these studies indicated a unique and bi-directional relationship between air pollution exposure and lung microbiome change.

**Table 4:** Animal studies performed on air pollution and microbial respiratory dysbiosis.

Study Population	Exposure	Sample Type	Results Summary
BALB/c mice, male [76]	PM_2.5_	Bronchoalveolar lavage	Shannon ↓, observed ASV ↓, Fisher ↓, weighted UniFrac (+)
C57BL/6 mice, male [77]	PM_2.5_	Lung tissue	Shannon ↓, Simpson ↓
C57BL/6N mice, male [81]	PM_2.5_	Bronchoalveolar lavage	(+) Serum and BALF: IL-1B, IL-6, IL-17, TNF-a, Simpson ↑, Shannon ↑, ACE ↑, Chao1 ↑, metabolic pathway alteration (+)
C57BL/6 mice, male [82]	Carbon black, ozone, CB + O_3_	Lung tissue	Neutrophils ↑, eosinophils ↑, Shannon ↓, total bacterial load ↓
Fischer 344 rat, male [83]	TRAP	Lung tissue	Lung function ↓: PEF, FVC, FEV
Sprague Dawley rats, male [80]	Biomass fuel, motor vehicle exhaust	Bronchoalveolar lavage	BALF macrophage ↑, IgA ↑, IgG ↓, Out ↑, Chao1 ↑, PD whole tree ↑, observed species ↑
C57BL/6 mice, male [78]	Diesel exhaust particle	Bronchoalveolar lavage	↑ BALF: IgA, IgG; ↑ lung: TNF-a, IL-10

↑ increase in index, ↓ decrease in index, (+) positive correlation to exposure.

From all of the studies, we can conclude that microbial alterations in response to different air pollutants are inconsistent, although *Staphylococcus*, *Hemophilus*, *Streptococcus*, *Neisseria*, *Moraxella*, and *Pseudomonas* are commonly altered. Changes in the microbiome depend upon several factors, including but not limited to pollutant characteristics, the route of exposure, host body site, and exposure time. Nevertheless, research on the impacts of air pollution on airway microbiome and disease progression requires more focus.

## 3. Gut Microbiome

The microbiome in the gut is associated with energy metabolism, nutrition, physiology, gut barrier integrity, immune function, etc. [84]. In the human GI tract, *Bacteroidetes* and *Firmicutes* are the most prominent, though more than 50 phyla have been identified [85]. Due to physiological and structural dissimilarities, different parts of the gut harbor diverse bacterial populations, e.g., *Bacteroides*, *Streptococcus*, *Enterococcus*, *Bifidobacterium*, *Lactobacillus*, *Clostridium*, and *Ruminococcus* are mostly found in feces, whereas only *Lactobacillus*, *Clostridium*, and *Enterococcus* are detected in the gut microbiome [86].

### Air Pollution and Gut Microbiome

The evaluation of the causal effect of air pollution (PM_2.5_, PM_10_, and NO_2_) indicated a significant correlation between particulate matter exposure and different diseases such as hypertension, obesity, and type 2 diabetes [23,87,88,89]. Gaseous or solid polycyclic aromatic hydrocarbon (PAH) exposure in 3 to 5-year-old children resulted in a positive correlation with a relative abundance of *Bacteroidetes*, *Actinobacteria* and *Proteobacteria* [90]. In another study, an increase in *Firmicutes* and *Bacteroides* was reported [91]. PM_1_ and PM_2.5_ exposure reduced the alpha diversity profile and showed a negative correlation with *Firmicutes*, *Proteobacteria*, and *Verrucomicrobia* abundance [88]. Another study on PM_2.5_ and PM_10_ showed no significant change in alpha diversity, although an increased abundance of *Bifidobacteriaceae*, *Porphyromonadaceae*, *Rikenellaceae*, and *Streptococcaceae* was observed [92]. Traffic-related air pollution (TRAP) reduced *Bacteroidaceae* and increased *Coriobacteriaceae* and was found to be correlated directly with the abundance of *Actinobacteria* and, inversely, with an abundance of *Proteobacteria* [93]. These studies showed that although the respiratory tract is the primary organ for pollution-mediated microbiome alteration, distal organs such as the gut can also be significantly impacted [83]. Human studies on the effects of air pollution on the gastrointestinal microbiome are presented in Table 5.

Studies in rodents reported that air pollution exposure, through inhalation or ingestion, can alter gut microbiota (Table 6). PM_2.5_ has been found to alter gut microbial diversity and induce an increased abundance in *Escherichia*, *Parabacteroides*, *Oscillibacter*, and *Akkermansia* [95]. Exposure to PM_2.5_ increased alpha diversity and beta diversity and is associated with an increased abundance of *Bacteroidetes* and a reduced abundance of *Firmicutes* [96]. Similarly, an increased abundance of Firmicutes and reduced Bacteroidetes, Cyanobacteria, and *Proteobacteria* was observed in Sprague Dawley rats exposed to PM_2.5_ [97]. This study also reported increased PM_2.5_ levels positively correlated with alpha and beta diversity alterations. A similar response was reported in Balb/c mice when exposed to PM_2.5_, although a decrease in the number of OTUs was observed [98]. In contrast, diesel exhaust particle (DEP) exposure in an apolipoprotein E knockout mice model resulted in a reduced Shannon and Simpson diversity index and increased the abundance of *Roseburia*, *Helicobacter*, and *Rikenellaceae* RC9 [99]. In another study, DEP exposure increased the abundance of *Firmicutes* and reduced *Proteobacteria* and *Actinobacteria* [79]. Similar to DEP exposure, exposure to concentrated ambient fine particulate matter (CAP) correlated with reduced alpha diversity, reduced *Clostridium*, *Papillibacter*, and *Turibacter*, and increased glucose intolerance and insulin resistance [100]. Distinct from particle exposure, gaseous exposure (nitric oxide) increased the abundance of *Sphingomonas*, *Actinomarina*, and *Bradyrhizobium* and decreased the abundance of *Rothia*, *Turibacter*, and *Corynebacterium* [101].

Taken collectively, all of these studies demonstrated an association between air pollution and gut microbiota; however, results vary greatly due to the varied composition of air pollutants, exposure route and duration, and the site of sample collection.

## 4. Potential Mechanism of Air-Pollution-Induced Respiratory Microbiome Dysbiosis

### 4.1. Alteration in Airway Physiological Environment

Exposure to air pollution alters the physiological environment of the airways, which is accompanied by an increase in oxidative stress and local inflammation accompanied by pH changes and local oxygenation [107,108]. Metabolites produced by microbes activate the NF-kB pathway through pattern recognition receptors to release pro-inflammatory cytokines. On the other hand, alveolar macrophages and T cells induce immune response by preventing bacterial overgrowth. Air pollution is reported to alter these interactions, thus leading to adverse effects on immune regulation [57,108]. Studies have also found that air pollution alters lactic acid, fumaric acid, and D-glyceric acid in the lungs, which are negatively correlated with the relative abundance of *Enterobacteriaceae*, *Ruminococcaceae*, and *Pseudomonadacea* [68,81].

### 4.2. Oxidative Stress

Oxidative stress responses can induce inflammation, cell death, and the generation of reactive oxygen species (ROS) and alter antimicrobial response [109]. PM contains organic and inorganic ROS-inducing components that can cause oxidative stress, leading to the destruction of epithelial and endothelial cells in the lungs and gut [110,111]. Free radicals generated by the RNS and ROS induce oxidation and damage in DNA strands and modify DNA repair proteins. Moreover, ROS also activate NFkB, which promotes epithelial–mesenchymal transition [112,113]. Cytokines (*IL-33*, *IL-25*, *TSLP*, and *GM-CSF*) produced by epithelial cells promote type 2 immune response by activating Th2 cells, macrophages, basophils, and eosinophils. Induced inflammatory response and increased ROS levels function in the disruption of cell–cell junctions, leading to the increased permeability of the epithelial barrier [114,115].

### 4.3. Disrupted Barrier Integrity

Epithelial barrier damage is one of the most common effects of air pollution. Airway epithelium acts as a physical and biological barrier between the outside environment and the intracellular compartment. It helps to maintain immune homeostasis and normal physiological functioning [116]. Epithelial barriers comprise three components: adherence junction, tight junction, and desmosomes. Pollutants disrupt barrier integrity by increasing inflammatory mediators and ROS, the dysregulation of transcription, and the expression of tight junctional markers like E-cadherin [117,118]. Studies have reported that PM can cause the degradation of junctional proteins via ROS production and impair lung function, facilitating bacterial invasion and dissemination in the blood [119]. Organic pollutants like PAH and inorganic materials (metals) in PM_2.5_ exposure have also been linked to inducing epithelial lesions by cytochrome p450 activation, the confluence of inflammatory cells, increased intestinal permeability, altered mucin production and secretion, and the decreased expression of the tight junctional protein, claudin-1, and desmosome [118,120,121]. Moreover, they also activated pathways (EGFR and ERK signaling), further destabilizing barrier integrity by disrupting tight junctions and adherence junctions. Pollutants also induced the release of amphiregulin and high-mobility group box 1 (HMGB1), which further promoted the breakdown of the barrier [122,123]. Therefore, the disruption of epithelial barrier integrity can result in altered immune homeostasis and induce inflammatory response, ROS production, microbial toxin, and LPS release in blood, thus leading to the initiation and aggravation of disease severity.

### 4.4. Disrupted Lipid Homeostasis and Systemic Inflammation

Lipids play an important role in regulating the inflammatory process through pro and anti-inflammatory lipid mediators. Alterations in lipid mediators have been linked to exacerbating systemic inflammation [124]. Zhivaki et al. [125] reported that air pollution is associated with elevated levels of blood lipid biomarkers, which potentially activate innate immune cells to initiate systemic pro-inflammatory response. The inflammatory response can also be induced by air pollution by facilitating the entry of pathogens into the systemic circulation. Such induction leads to the production and systemic release of inflammatory cytokines (*TNF-alpha*, *IL-1b*, and *IL-6*) [126]. This cumulatively contributes to the aggravated loss of barrier function, thus inducing systemic inflammation in distant organs [127].

In sum, the mechanisms that disrupt the microbiome in the lungs (and in the gut) are centered around central processes of redox imbalance and inflammation. These processes create changes in airway physiology and homeostasis, leading to an altered microenvironment in the lung airways and parenchyma that facilitate a disease phenotype. In the case of the gut, the central process seems to be altered intestinal barrier integrity, which facilitates the systemic manifestations of microbial dysbiosis.

## 5. Lung–Gut–Liver Axis

**Figure 2:**
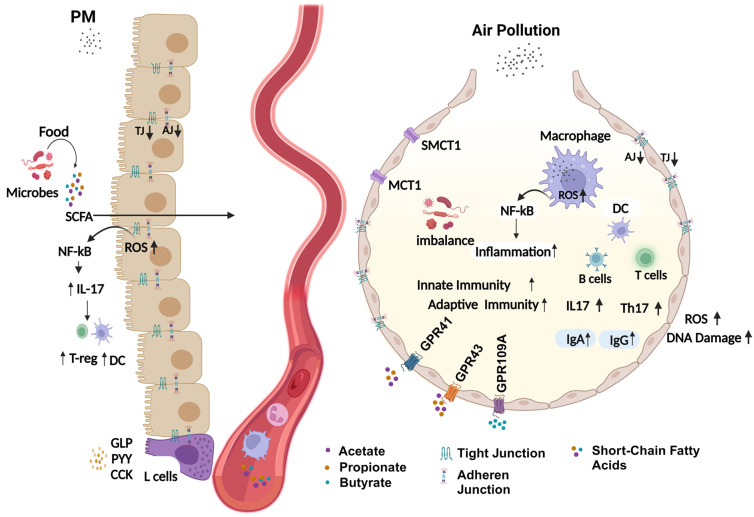
Mechanism of air-pollution-mediated microbial alteration and inflammation. In the healthy state, short-chain fatty acids maintain immune homeostasis by inducing the production of anti-inflammatory mediators and balance between immune cells. In the case of air pollution, excessive production of ROS disrupts epithelial barriers, production of pro-inflammatory mediators (*IL-17*), and subsequent production of IgA and IgG, and increased NF-kB activity leads to a significant shift in innate and adaptive immunity. Gut-derived altered secondary metabolite production (*CCK*, *GLP*, and *PYY*) and SCFA imbalance reduce barrier integrity and increase T_reg_ and DC infiltration. Acetate, propionate, and butyrate, through binding to lung receptors, modulate lung microbiome changes. Moreover, reduced expression of *MCT1* and *SMCT1* (transmembrane proteins) leads to decreased diffusion, thus increasing the accumulation of inflammatory mediators.

The lung–gut–liver axis pertains to the sequence of events in the lungs, gastrointestinal tract, and liver in conjunction with their role in the immune system, microbial regulation, and metabolic reaction [38,128]. The impacts of air pollutants on the microbiome can be direct or indirect. Air pollutants reach the gut mainly through ingestion to modify the microbiome [96,129]. Some active processes that follow the pulmonary deposition of air pollutants, such as an inflammatory response, redox imbalance, alteration in the airway microenvironment, disruption in air–blood barrier integrity, and changes in pulmonary lipid homeostasis can impact microbiome composition locally in the lungs and systemically. As such, the production of microbiota-derived signaling molecules (LPS, short-chain fatty acids—SCFAs) is altered in response to air pollution exposure, which disturbs immune response. Studies have found that *Bacteroides* spp. produced LPS-activated immune responses and were associated with autoimmune disease progression [130]. In addition, altered circulatory levels of microbiota-derived compounds can lead to systemic inflammation and distal organ dysfunction [131]. In the healthy state, SCFAs and microbe-associated molecular patterns (MAMPs) derived from gut microbiota maintain immune homeostasis by generating anti-inflammatory mediators, regulatory T cells, and IgA [132,133]. SCFAs and Toll-like receptors (TLR) together support the generation of tight junction proteins, which are important to maintain barrier integrity [134]. Moreover, G-protein coupled receptors (GPCRs) recognize SCFAs in the lungs, leading to anti-inflammatory effects and the regulation of epithelial cellular integrity in the lungs [37]. G proteins mediate their effects through the activation of mitogen-activated protein kinase (MAPK), phosphoinositide 3 kinase (PI3K), and mTOR [135]. Another mechanism of SCFA’s modulation of immune response is through the inhibition of histone deacetylase (HDAC). Moreover, SCFAs enter cells via passive diffusion or absorption and exert a suppressive effect through SLC5A8 (high-affinity Na^+^-coupled monocarboxylate transporter) or low-affinity H^+^ couple carrier SLC16A1 [87,136]. Air-pollution-induced alterations in microbial composition, SCFAs, MAMP levels, and ROS production induce pro-inflammatory response, disrupt lipid homeostasis, and destroy epithelial integrity, leading to barrier function loss (Figure 2) [37,129]. *Bacteroides fragilis*-derived polysaccharide A induces *IL-10* production by T cells and protects chemically induced intestinal inflammatory response [137]. Moreover, some bacteria produce cell membrane components (e.g., sphingolipids) that decrease the number of natural killer T cells, which have been correlated with the development of colitis [138].

**Figure 3:**
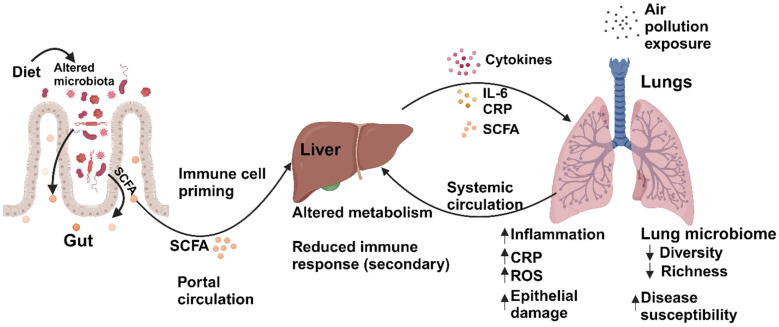
Gut–liver–lung’s axis regulation alteration by the microbiome. Factors such as diet, pollution, and antibiotics alter the gut microbiome, which produces SCFAs and other secondary metabolites. These metabolites travel through the portal vein and induce activation of macrophages and neutrophils. Induced cytokines, immune regulators, and secondary metabolites through systemic circulation regulate ROS generation, CRP production, and epithelial damage in the lungs. These also led to alterations in the diversity and richness of the lung microbiome. Microbiome change in the respiratory tract caused by air pollution also modulates the alteration of microbial balance in the gut through the lung–gut–liver axis.

The gut microbiota depletion induced a significant increase in inflammatory response in the lung and a 30% increase in mortality in a pneumonia mouse model [139]. The gut microbiome increases lung inflammatory cell infiltration against bacterial challenge and induces neutrophil accumulation through TLR4 [140]. The depletion of the gut microbiome was found to reduce *TGF-b* and granulomas in the lung, indicating gut–lung communication and the role of microbial dysbiosis in disease pathology. This study also indicated gut alpha and beta diversity shifted due to carbon nanotube exposure in the lungs, showing a bidirectional relation [106]. Studies have also found a link between changes in gut and lung microbiome and liver metabolism alteration. This bidirectional communication integrates dietary, genetic, and environmental signals [141]. The dysregulation of gut microbiota can cause hepatic inflammation [142]. In the case of chronic liver disease and hepatic fibrosis, gut dysbiosis leads to reduced hepatic anti-inflammatory mechanism by increased TLR4 activation through the TGF-beta signaling pathway [143,144]. Bile acids derived from the liver function as a metabolic health gatekeeper can also regulate the composition and community profiling of the gut microbiome [145]. Similarly, the liver–lung axis is also reported to have a significant correlation [146,147,148]. All of these suggest that the lung, gut, and liver orchestrate a microbiome-mediated production of secondary substances to regulate immune-modulatory functions [128]. An overview of this pathway is presented in Figure 3.

## 6. Microbiome as a Therapeutic Approach and Target

The alteration of the microbiome is a powerful and innovative tool that can improve healthcare efficiency. Complementary biotic approaches (probiotics, prebiotics, and synbiotics) and fecal microbiota transplantation have been proposed to manipulate the microbiome [149]. Studies have shown that microbe-based therapies can alleviate the pathophysiological effects of allergic airway disease, ulcerative colitis, insulin resistance, type 2 diabetes, etc. [150,151]. Although promising results are observed in microbiome-based therapeutic approaches, there are several facets to altering bacterial community. These can be broadly combined into three categories: (1) the selection of the microbiome, (2) the analysis of the microbiota to harbor in a specific niche, and (3) balance between residing and newly introduced microbiomes [152]. First, the microbiome of healthy individuals serves as a prototype for selecting an ideal microbiome though age, sex, and epigenetic background have a strong role to play. Secondly, the recent advancement in sequencing and metabolomics helps to identify shifts in microbiome clustering patterns among healthy and diseased individuals [153]. Microbiome composition varies significantly based on the anatomical location; thus, the molecular and metabolic analysis of region-specific microbiome is important. Finally, the modified microbial community interacts with residing microbes and competes to adapt to the host environment. Thus, multi-omics comparisons are required to analyze hundreds of potential community interactions.

## 7. Conclusions and Future Perspectives

The influence of the microbiome on the host depends not only on the health status of the host but also on the microbiome function and composition, metabolites released by microbes, and local and systemic innate and adaptive immune responses of the host. The impact of air pollution exposure varies depending on pollutant composition, the origin of pollutants, exposure route, and host health status. Compelling evidence demonstrates a correlation between microbiome alterations and impaired lung function, lung development, immune modulation, and barrier function, especially in different diseases. While the literature points towards a strong association between microbial dysbiosis and air pollution exposure, further research should focus on whether dysbiosis is a cause of exposure-induced pathology or its consequence. An interesting unanswered question is whether environmental exposures have a unique dysbiosis signature or whether different pollutant exposures can be grouped based on their dysbiosis signature to streamline intervention approaches. Although this review describes potential interactions between the lung and gut microbiome, further research is warranted to elaborate mechanistic pathways. Overall, looking at the current reported literature, we identify that while this is an expanding field of research that has yielded very valuable insights into different physiological and pathological processes, studies reporting on microbiomes are not following a consensus approach for performing and reporting experimental findings. Several shortcomings that need to be addressed in future studies concerning microbiome and air pollution include the use of relevant models for exposure (e.g., realistic inhalation exposure), the use of environmental samples (e.g., PM_2.5_ collected from the air), the complete identification/reporting of not just bacterial but also viral and fungal microbiomes, reporting species level changes, and the need to go beyond the association of different exposures and changes in microbiome to causality relationships. In addition, clinical trials evaluating microbiome homeostasis and SCFA-mediated regulation can be of future importance. Finally, another critical need is to establish cellular mechanisms and processes that are directly vs. indirectly impacted by the microbiome. These studies are essential to establish the contributing role of the microbiome in the pathophysiology of different diseases and will enable the development of potential therapeutic strategies.

## Figures and Tables

**Table 1:** Dominant bacterial community profiles in various body sites (arranged in descending order).

Body Site	Dominant Bacterial Communities
Skin	*Corynebacteria*, *Propionibacteria*, *Staphylococcus*, *Streptococcus*, *Moraxella*, *Dolosigranulum*
Oral cavity	*Prevotella*, *Veillonella*, *Streptococcus*, *Corynebacteria*, *Neisseria*, *Haemophilus*, *Fusobacterium*, *Rothia*
Lung	*Prevotella*, *Streptococcus*, *Haemophilus*, *Fusobacterium*, *Actinobacteria*
Gut	*Firmicutes*, *Bacteroidetes*, *Actinobacteria*, *Proteobacteria*, *Fusobacteria*
Urogenital tract	*Prevotella*, *Gardnerella*, *Atopobium*, *Lactobacillus*, *Escherichia*, *Enterococcus*, *Shigella*, *Streptococcus*, *Citrobacter*

**Table 2:** Lung microbiota differs among healthy individuals and respiratory disease patients.

Condition	Taxa (Major Genera)
Healthy [53]	*Veillonella*, *Fusobacterium*, *Prevotella*, *Streptococcus*, *Porphyromonas*, *Neisseria*
Asthma [54]	*Haemophilus*, *Streptococcus*, *Prevotella*, *Klebsiella*, *Moraxella*
COPD [55,56]	*Moraxella*, *Streptococcus*, *Haemophilus*, *Streptococcus*, *Pseudomonas*
Lung cancer [57,58]	*Streptococcus*, *Abiotrophia*, *Granulicatella*, *Veilonella*, *Staphylococcus*, *Haemophilus*
Cystic fibrosis [59,60]	*Streptococcus*, *Prevotella*, *Veillonella*, *Gemella*, *Neissera*, *Rothia*, *Actynomyces*, *Haemophilus*
Idiopathic pulmonary fibrosis [61,62]	*Hemophilus*, *Neisseria*, *Streptococcus*, *Staphylococcus*, *Veillonella*

**Table 3:** Human studies performed on microbial dysbiosis and air pollution.

Study Population	Exposure	Sample Type	Results Summary
Healthy and COPD Volunteers [70]	PM_2.5_	Sputum sample	Higher FEV_1_/FVC ratio to bacterial load (+), OTU (+)
Farmer’s Market Vendors [69]	PM_2.5_−200 ug/m^3^, PM_10_−300 ug/m^3^	Pharyngeal swabs	Chao1 ↑, ACE ↑, correlation with microbiome: smog (+), gender (+), smoking (+), mask (−)
Healthy Volunteers [71]	PM_2.5_, PM_10_	Nasal swab	Shannon (−), Chao1 (−), PD whole tree (−)
Asthmatic Children [73]	PM_2.5_ or ozone	Broncho alveolar lavage	Species richness (−), observed species (−)
Young Adults [74]	PM_2.5_	Sputum	Cytokine ↑: IL4, IL6, IL17, TNF−a, IFN−g
Healthy Volunteers [65]	PM_2.5_, PM_10_, NO	Throat swab	Lung function ↓
Lung Cancer Patients [72]	PM_10_	Lung tissues	PD whole tree (+)
Children [64]	Traffic-related air pollution	Saliva and sputum	Shannon ↑, observed ASV ↑, phylogenetic diversity ↑
Healthy and Lung Cancer Females [67]	Household air pollution	Sputum	Alpha diversity (−), observed species (−), unweighted UniFrac (+)
Healthy Subjects [66]	Household air pollution	Broncho alveolar lavage	No alpha, beta diversity change
Adults [75]	Indoor dust	Nasopharyngeal swabs	ASV, Shannon
Healthy Young Adults [68]	O_3_−200 ppb; 2 h	Nasal secretion	Serum CC16 ↑, FEV_1_ ↓, FVC ↓, glucose ↑, lactic acid ↑, D−glyceric acid ACE ↓, Simpson ↓, Shannon ↓, weighted UniFrac (#)

↑ increase in index, ↓ decrease in index, # distinct difference present, (+) positive correlation to exposure, (−) negative correlation to exposure.

**Table 5:** Human studies performed on air pollution and GI tract microbiome alterations.

Study Population	Exposure	Sample Type	Results Summary
Children between ages 3 and 5 [90]	Air PAH level	Soil, stool, skin	PPAR (+), adipocytokine signaling pathway (+)
Young adults [88]	PM_2.5_, PM_1_	Stool	Type 2 diabetes (+), Shannon (−), Chao1 (−), PD whole tree (−)
Adults [94]	PM_2.5_	Stool	Shannon ↓
Children [92]	PM_10_, PM_2.5_, smog	Gut	No Shannon and Chao1 index difference No weighted and unweighted UniFrac change
Adults [93]	Traffic-related air pollution, nitrogen oxides	Gut	Impaired glucose homeostasis
Young adults [91]	Air pollution	Gut	Shannon ↑

↑ increase in index, ↓ decrease in index, (+) positive correlation to exposure, (−) negative correlation to exposure.

**Table 6:** Studies performed on air pollution and microbiome alteration in animals.

Study Population	Exposure	Sample Type	Results Summary
C57BL/6J mice, male [96]	PM_2.5_	Gut and gut content	↑ Feces: observed OUT, Chao1, PD whole tree, unweighted UniFrac (+), Bray–Curtis similarity (+)
BALB/c mice, male [98]	PM_2.5_	Gut	IL-6 ↑, IL-8 ↑, TNF-a ↑, OTU ↓, Chao1 ↑, Shannon↑
C57BL/6 mice, male [82]	CB, O_3_ and CB + O_3_	Fecal content	Total bacterial load ↑, SCFA: acetate ↑, propionate ↑
Sprague Dawley rats, male [97]	PM_2.5_	Stool	Shannon ↑, Chao1 ↑, Simpson ↓, ACE ↑, weighted UniFrac (+)
C57BL/6 mice, male [102]	Ultra-fine particles	Fecal content	ASV ↑, Shannon ↑
Ldr KO mice [103]	Ultra-fine particles	Gut content	Chao1 ↓, Faith PD ↓, Shannon ↓, unweighted UniFrac (+), weighted UniFrac (+), ↑plasma: TNF-a, MCP-1, LPC18:1
C57BL/6J mice, male [100]	Concentrated ambient particle	Feces	Glucose intolerance (+), insulin resistance (+), ACE ↓, Chao1 ↓
C57BL/6 mice, female [99]	Diesel exhaust particle	Gut content	Shannon ↓, Simpson ↓, weighted UniFrac (+) Cecal SCFA ↓, triglycerides ↓
C57BL/6 mice, male [79]	Diesel exhaust particle	Gut content	Chao1 ↓, ACE ↓, plasma LPS ↑, IL-13 ↑, G-CSF ↑, MIP-2 ↑, TNF-a ↑
C57BL/6J mice, male [95]	Diesel exhaust particle	Feces	OUT ↓, Chao1 ↓, Shannon ↓, Goods coverage ↑, unweighted UniFrac (+)
BABL/c mice, female [104]	House dust	Gut content	Fast UniFrac (+), ↓ lung: IL-13, IL-4
C57BL/6 mice, male [105]	Cigarette smoke	Gut content	Shannon ↓, Muc5b ↑, Muc4 ↓
C57BL/6 mice [106]	Carbon nanotube + cigarette smoke	Feces	Shannon ↑, Chao1 ↑, total protein content ↑, CXCL1 ↑, TGF-beta ↑
Sprague Dawley rats, male [101]	Ambient NO_2_	Gut	PD whole tree ↑, unweighted UniFrac (+), cardiac mfn2 ↓, HSP70↓

↑ increase in index, ↓ decrease in index, (+) positive correlation to exposure.

## Data Availability

No new data was created, and data sharing does not apply to this article.

## Abbreviations

AJ—Adherent junction, ARDS—Acute respiratory distress syndrome, ASV—Amplicon sequence variants, ARI—Acuter respiratory infection, BALF—Bronchoalveolar lavage fluid, CAP—Concentrated ambient particulate matter, CCK—Cholecystokinin, CF—Cystic fibrosis, CFTR—Cystic fibrosis transmembrane conductance regulator, COVID—Corona virus disease, COPD—Chronic obstructive pulmonary disease, DC—Dendritic cells, DEP—Diesel exhaust particle, FEV—Forced expiratory volume, FMT—Fecal microbiota transplantation, FOS—Fructo oligosaccharide, FVC—Forced vital capacity, GI—Gastrointestinal tract, GLP—Glucagon like peptide, GOS—Galacto oligosaccharide, GPCR—G-protein coupled receptor, HMP—Human microbiome project, IBD—Inflammatory bowel disease, IBS—Irritable bowel syndrome, IL—Interleukin, IPF—Idiopathic pulmonary fibrosis, LPS—Lipopolysaccharide, LRT—Lower respiratory tract, MAMP—Microbe associated molecular pattern, MCT1—Monocarboxylate transporter1, NGS—Next generation sequencing, PAH—Polycyclic aromatic hydrocarbon, PM—Particulate matter, ppb—parts per billion, ROS—Reactive oxygen species, SCFA—Short chain fatty acid, SMCT1—Sodium coupled monocarboxylate transporter-1, TJ—Tight junction, TLR—Toll like receptor, TRAP—traffic-related air pollution, UC—Ulcerative colitis, URT—Upper respiratory tract, WHO—World Health Organization.
